# Analysis of Leakage Current of HfO_2_/TaO_*x*_-Based 3-D Vertical Resistive Random Access Memory Array

**DOI:** 10.3390/mi12060614

**Published:** 2021-05-26

**Authors:** Zhisheng Chen, Renjun Song, Qiang Huo, Qirui Ren, Chenrui Zhang, Linan Li, Feng Zhang

**Affiliations:** 1 School of Electronic and Information Engineering, Beijing Jiaotong University, Beijing 100044, China; chenzhisheng@ime.ac.cn (Z.C.); 18125048@bjtu.edu.cn (R.S.); 14213033@bjtu.edu.cn (C.Z.); 2 Key Laboratory of Microelectronics Device and Integrated Technology, Institute of Microelectronics of the Chinese Academy of Sciences, Beijing 100029, China; huoqiang@ime.ac.cn (Q.H.); renqirui@ime.ac.cn (Q.R.)

**Keywords:** resistive random access memory (RRAM), 3-D integration, self-selective cell (SSC), sneak path, leakage current

## Abstract

Three-dimensional vertical resistive random access memory (VRRAM) is proposed as a promising candidate for increasing resistive memory storage density, but the performance evaluation mechanism of 3-D VRRAM arrays is still not mature enough. The previous approach to evaluating the performance of 3-D VRRAM was based on the write and read margin. However, the leakage current (LC) of the 3-D VRRAM array is a concern as well. Excess leakage currents not only reduce the read/write tolerance and liability of the memory cell but also increase the power consumption of the entire array. In this article, a 3-D circuit HSPICE simulation is used to analyze the impact of the array size and operation voltage on the leakage current in the 3-D VRRAM architecture. The simulation results show that rapidly increasing leakage currents significantly affect the size of 3-D layers. A high read voltage is profitable for enhancing the read margin. However, the leakage current also increases. Alleviating this conflict requires a trade-off when setting the input voltage. A method to improve the array read/write efficiency is proposed by analyzing the influence of the multi-bit operations on the overall leakage current. Finally, this paper explores different methods to reduce the leakage current in the 3-D VRRAM array. The leakage current model proposed in this paper provides an efficient performance prediction solution for the initial design of 3-D VRRAM arrays.

## 1. Introduction

Due to the high endurance, high nonlinearity, and robust read/write disturbance immunity [1,2,3], resistive random access memory (RRAM) has received enormous attention as one of the most promising candidates for the next generation of nonvolatile data storage technology [4,5,6,7]. Different from the traditional charge-type memory, the read and write operations of the RRAM are significantly affected by circuit-level factors such as the working mode and interconnection [8,9,10,11]. To further increase the storage density of resistive random access memory, the 3-D VRRAM architecture is proposed, which increases the storage density by stacking RRAM cells in the vertical direction. Most of the current research about 3-D VRRAM are based on the single memory cell level [12,13,14,15,16,17]. Therefore, it is significant to estimate the performance of 3-D VRRAM at the array level [18,19]. Recently, the read and write margin of 3-D VRRAM with a WL planar structure has been evaluated in a few papers [20,21]. However, many opinions believe that the interconnection sneak path in the 2-D and 3-D architectures is the limiting factor for establishing large-scale RRAM arrays [22,23,24]. The reliability, array expansibility, and array read-write accuracy of RRAM decreases significantly with the increase of the leakage current in the RRAM array [25]. A high leakage current generates additional power consumption, reducing the energy efficiency ratio of the system [26]. The establishment of a leakage current model with excellent characteristics can help in the early design of RRAM chips. However, the leakage current has not been fully analyzed in previous work.

This research proposes a direction for the design and the selection of the read/write scheme in 3-D VRRAM arrays by analyzing the leakage current (LC). The remainder of this paper is organized as follows. In Section 2, we presented the architecture of 3-D VRRAM, analyzed the cause of leakage current, and described the voltage bias scheme used in the simulation. In Section 3, the SPICE simulation results are shown, and the factors that affect the leakage current in the RRAM array are analyzed. Finally, Section 4 concludes this article.

## 2. Simulation Methods

**Figure 1:**
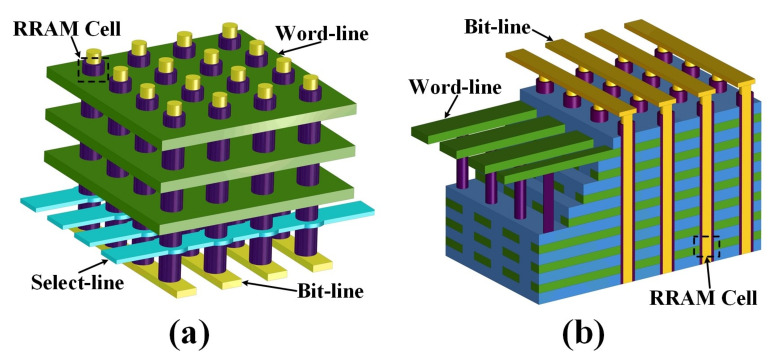
(**a**) Schematic of traditional 3-D VRRAM and (**b**) schematic of HfO${}_{2}$/TaO${}_{x}$-based on a built-in nonlinear 3-D VRRAM.

Figure 1a illustrates the schematic of traditional 3-D VRRAM. This architecture uses word lines (WL), select lines (SL), and bit lines (BL) to select RRAM cells in the array. WLs are plane electrodes that intersect the pillar’s electrode. SLs are used to choose the target column in the array. Moreover, the different pillars are also connected by BLs at the bottom of the array.

**Figure 2:**
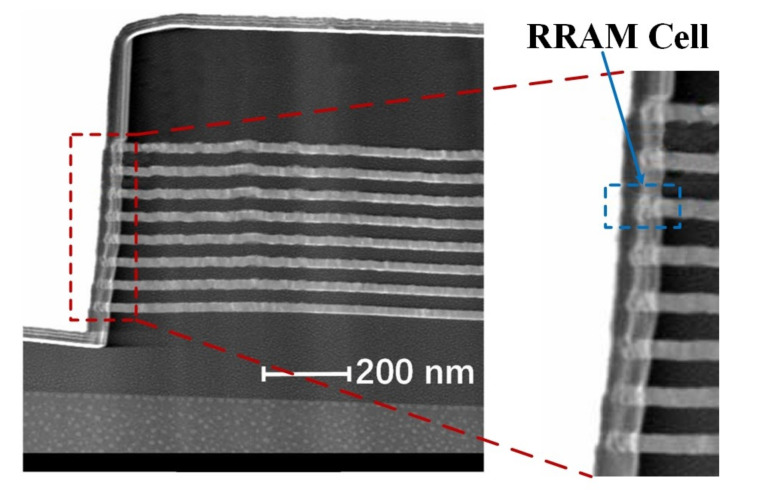
TEM image of the HfO${}_{2}$/TaO${}_{x}$-based built-in nonlinear 3-D VRRAM structure.

In this study, we propose another HfO${}_{2}$/TaO${}_{x}$-based built-in nonlinear 3-D VRRAM (BNR) architecture [27]. Due to the institution of a high-performance self-selection cell (SSC), the architecture only contains bit-lines and word-lines, as shown in Figure 1b, which can achieve higher circuit efficiency and operation margins. The transmission electron microscope (TEM) image of the built-in nonlinear 3-D VRRAM structure is depicted in Figure 2. The resistance of 2-D RRAM is changed with the change in the conductive filament (CF). However, the mechanism of the resistance of the memory cell in 3-D VRRAM array is different. Under the action of the electric field, the vacancies in the barrier layer shift under different bias voltages, and the width of the tunnel barrier change accordingly, thereby changing the resistance of the RRAM device.

**Figure 3:**
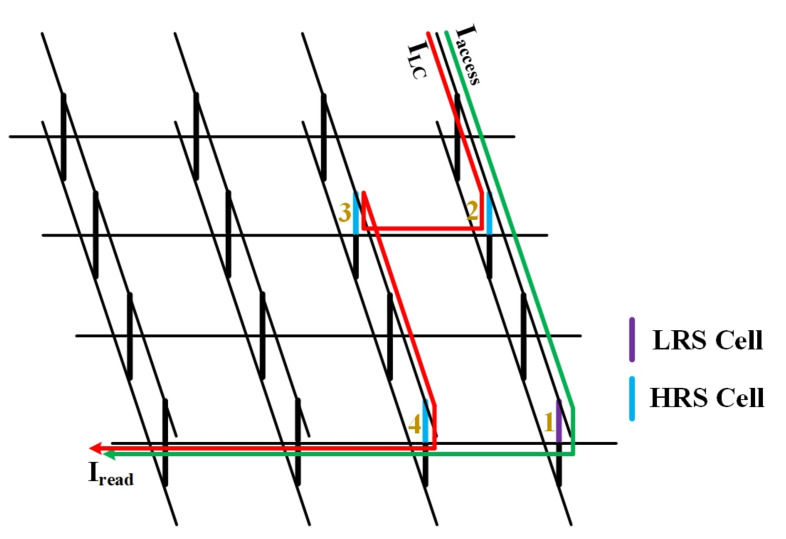
Sneak path in 3-D VRRAM array.

To facilitate this demonstration, we use a schematic diagram of the 3-D VRRAM array to explain the cause of the leakage current, as shown in Figure 3. When the No. 1 device is selected, if the status of the No. 1 cell is HRS and the No. 2 to No. 4 cells are in the LRS status, the black line indicates a complete current loop. However, the current also flows through the No. 2 to No. 4 cells, as indicated by the red line, forming a sneak path. As is well known, more sneak paths produce greater leakage current.

**Figure 4:**
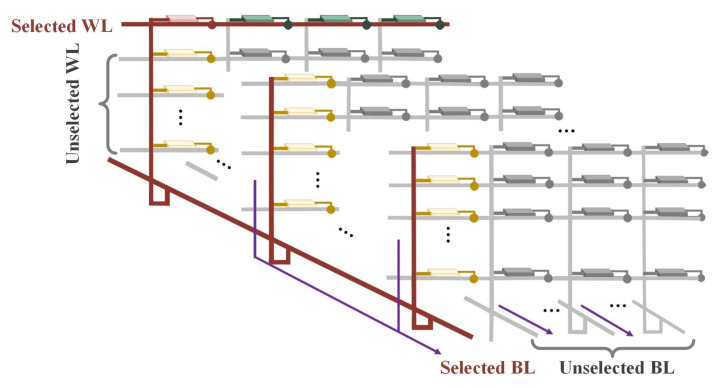
Spice model of the novel 3-D VRRAM array.

Compared with the 2-D RRAM array [28,29], there are more sneak paths in the 3-D RRAM array, so the leakage current in the 3-D VRRAM array passes through more RRAM cells, resulting in greater additional energy loss. Therefore, it is important to evaluate the leakage current in the early stages of 3-D VRRAM array design. The complete nonlinear *I-V* characteristics of the RRAM device are not included in the SPICE simulation because including them greatly reduces the simulation speed and requires more memory resources to evaluate the performance of enormous array size. However, compared with other work that used the simple analytic approximation model for the array investigation [30,31], our HSPICE simulation method based on modular analysis is more accurate. We developed a 3D circuit module, as shown in Figure 4 for the HSPICE simulation. The model proposed in this article can be divided into four parts: the red resistor represents the selected RRAM cell; the green resistors represent half-selected RRAM cells on the same WL; the yellow resistors represent half-selected RRAM cells on the same BL; and the gray resistors represent unselected RRAM cells.

**Figure 5:**
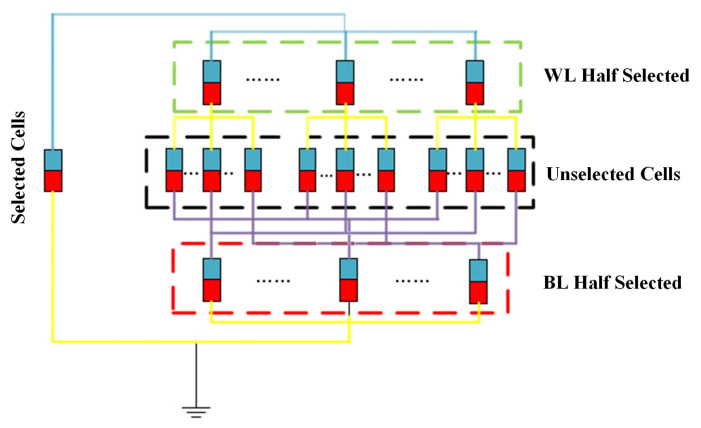
Leakage current model of the 3-D VRRAM array.

To analyse the leakage current of the 3-D VRRAM array, the model is simplified to the circuit model shown in Figure 5. The left side shows the current path of the selected RRAM cell, and the right side shows the sneak path that generates the leakage current.

**Figure 6:**
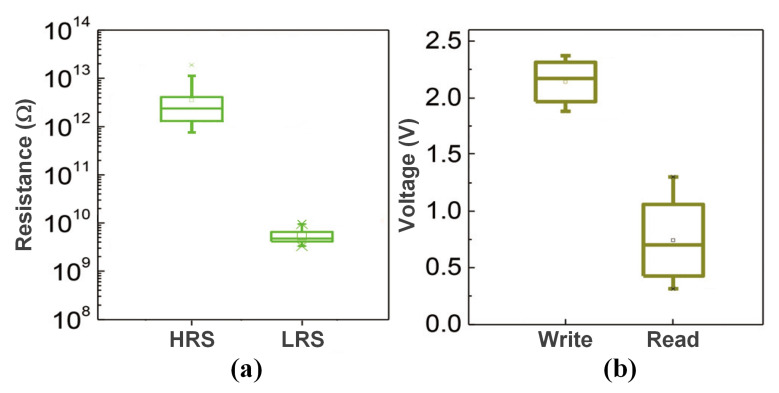
(**a**) Resistance distributions and (**b**) voltage distributions of 50 BNR devices.

Compared with other reported 3-D VRRAM structures, the structure we present in this paper has a higher resistance (the resistance levels of HRS and LRS are $10^{12}\;\Omega$ and $10^{9}\;\Omega$, respectively), as shown in Figure 6a. The wire resistance in the array is less than 10 Ω, the voltage drop caused by the wire resistance is tiny. Consequently, the influence of the wire resistance can be ignored in the analysis before the array size reaches 1 Gb. Moreover, the high resistance of the RRAM devices can ensure an outstanding read and write margin in a large array. Therefore, in this structure, the leakage current is the factor that requires more consideration in the array design compared to the operation margin.

We analyze the 1-bit operation scheme first and discuss the multi-bit operation scheme later in this article. In the 1-bit write operation, only one WL and one BL are selected to choose the selected device, and the other lines are unselected. The applied voltages at the selected and unselected WLs and BLs for read and write operations are listed in Table 1. For a read operation, a voltage of $V_{r}$ is applied to the selected word line, while all other lines are “0”. During the write operation, the WL and BL voltages of the selected RRAM cell are set to $V_{w}$ and 0, respectively, and all of the other lines apply a voltage of $V_{w}$/2 to prevent accidental writing.

**Figure 7:**
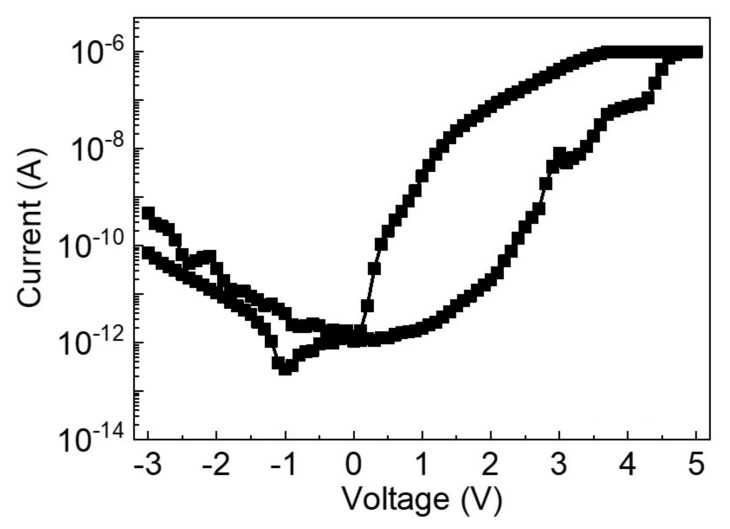
*I-V* characteristics of 3-D VRRAM cell.

The *I-V* characteristic of the HfO${}_{2}$/TaO${}_{x}$ based built-in nonlinear 3-D VRRAM architecture is shown in Figure 7. It can be seen from the figure that, when the compliance current is set to 1 µA, the resistance switching window of the device is still very large, which proves that it can normally work at currents of nA level. Compared with many RRAM devices that need to work at µA currents, this device has the advantage of low power consumption. Therefore, the leakage current must be strictly limited to avoid additional power consumption and to maintain its low power consumption characteristics. It is worth mentioning that, although the scan loop of the voltage is 0 V to 5 V, this does not indicate that the device requires a 5 V write voltage. In fact, a pulse voltage of approximately 2.5 V is sufficient to write to the device. Although the resistance ratio on the negative current range is lower than the positive range, this does not affect the switching mode of the device because all of the cells in the array are read in the positive range. When designing the peripheral circuit, the designer only needs to pay attention to the window that displays the forward curve.

The read and write voltage distribution of the novel BNR cell is shown in Figure 6b. It can be seen from the Figure that the range of the write voltage is 1.8–2.5 V, so $V_{w}$ (write voltage) is set to 3 V in this simulation to obtain a 0.5 V liberality, while making $V_{w}$/2 = 1.5 V to avoid intrusion to the half-selected area. To explore the effect of the read-voltage on the leakage current, $V_{r}$ (read voltage) is set to 1–1.5 V. Furthermore, we use the worst cell patterns proposed in Table 2 to analyze the worst-case leakage current [32].

The leakage current (LC) is defined as the total leakage current from all sneak paths

(1) $$LC=I_{whs}+I_{bhs}+I_{us}$$

where $I_{whs}$ denotes the leakage current of the half-selected area that shares the same WL with the selected cell. $I_{bhs}$ represents the leakage current of the half-selected area of the same BL as the selected cell, and $I_{us}$ represents the leakage current of the unselected area. Read Margin (RM) is defined as the difference between the current when the RRAM cell is in a low resistance state and when it is in a high resistance state, as shown in the following equation.

(2) $$RM=I_{LRS}-I_{HRS}$$

## 3. Results and Discussion

### 3.1. Error Rate

To analyze the leakage current in the 3-D VRRAM array, we performed numerous HSPICE simulations for different factors by controlling the variables. In order to verify the correctness of the simulation model proposed in this paper, we compared the simulation results with the experimental results of the RRAM array and defined the *Error-Rate (ER)*

(3) $$ER=\frac{|I_{lc\underline{\;\;}exp}-I_{lc\underline{\;\;}sim}|}{I_{lc\underline{\;\;}exp}}\times 100\;\left[\%\right]$$

where $I_{lc\underline{\;\;}exp}$ expresses the leakage current measured by experiments and $I_{lc\underline{\;\;}sim}$ represents the leakage current obtained by simulation. The maximum error does not exceed 0.7% when the array size is 32×8×8 and $V_{r}$ = 1 V, which shows the simulation results are in good agreement with the experimental results.

### 3.2. Array Size

This section discusses the comprehensive effects of the number of layers and the plane size of the array on the leakage current in both read and write modes.

**Figure 8:**
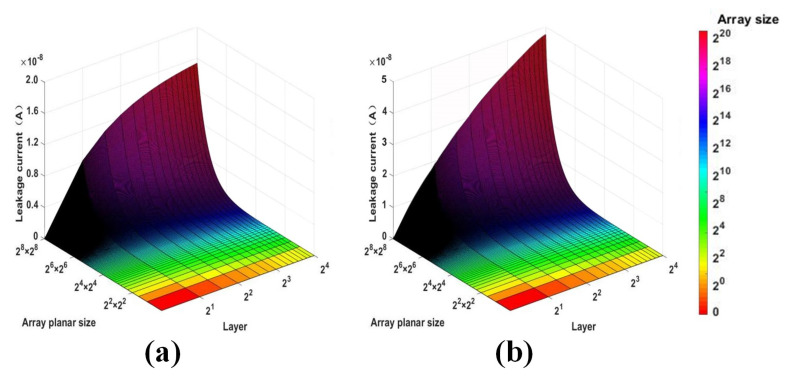
(**a**) Read leakage current under different array sizes and (**b**) write leakage current under different array sizes (from 4×4 to 256×256 and 1~16 layers).

With a fixed $V_{r}$ of 1 V, the leakage current of 3-D VRRAM array in various planar array sizes (4×4~256×256) and layers (1~16) is shown in Figure 8a. It can be seen from the figure that the leakage current is increased with the size of the planar array. Moreover, the leakage current is more obviously affected by the size of the planar array as the number of layers increases. This is because a larger number of layers corresponds to a higher growth rate of the sneak path. Figure 8b shows the relationship between the leakage current and the array size during a write operation. As with the read operation, as the size of the planar array and the number of layers increases, the leakage current of the write operation also increases significantly, and the leakage current of the write operation is higher than that of the read operation. This occurs because, compared to the read operation, the write operation applies a higher voltage. As shown earlier, the voltage has a significant influence on the leakage current. Therefore, it is necessary to balance the number of layers and the size of the planar array to minimize the leakage current when designing the apparatus.

### 3.3. Read Voltage

The maximum read voltage can approach half of the write voltage to prevent any storage state interference. Previous studies have shown that the read margin of the RRAM array increases as the read voltage rises. Nevertheless, in the experiment, we found that, as the read voltage increases, the leakage current of the 3-D RRAM array also increases and that excessive leakage current cannot be tolerated when designing the 3-D VRRAM array. Therefore, before designing a 3-D VRRAM array, the impact of operating voltage on leakage current must be evaluated to determine the operating voltage of the array. This section discusses the relationship between reading margin, leakage current, and read voltage in detail.

**Figure 9:**
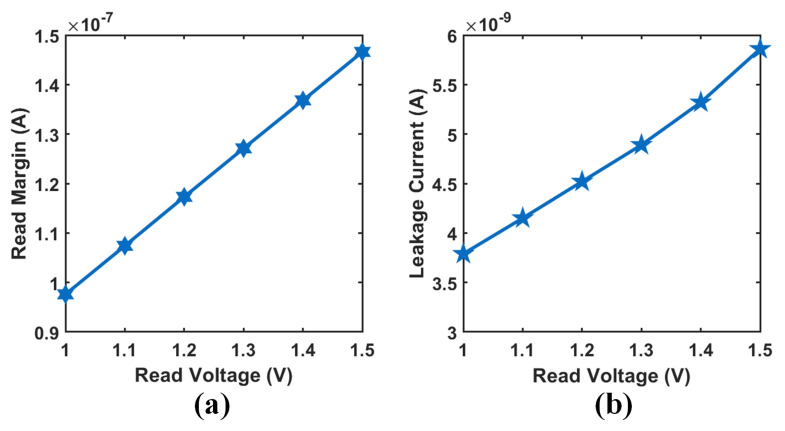
(**a**) RM under different read voltages and (**b**) LC under different read voltages.

Figure 9 shows the curve of RM and LC under different read voltages when the array size is 64×64×8. With the increase in the read-voltage from 1 V to 1.5 V, although the read margin is increased, the corresponding leakage current also increases about 2.1 nA, indicating that the additional energy consumption of the array increases. Although a lower read voltage reduces the overall leakage current and power consumption, it also significantly reduces the read margin of the 3-D VRRAM array and may result in an excessively small read current. This might bring a great challenge to read comparison and sensitivity amplifiers. Therefore, a balance between leakage current and read margin is considered during the 3-D VRRAM array design.

Comparing the effect of the array size and the read voltage on the leakage current of the memory, it can be found that the read voltage has a greater effect on the leakage current compared to the array size. This is because, for the 3-D VRRAM array, the read voltage is a global variable that affects all memory cells, and the expansion of the array size causes the sneak path to increase so that the read voltage has a greater impact on the leakage current.

### 3.4. Multi-Bit Operation

**Figure 10:**
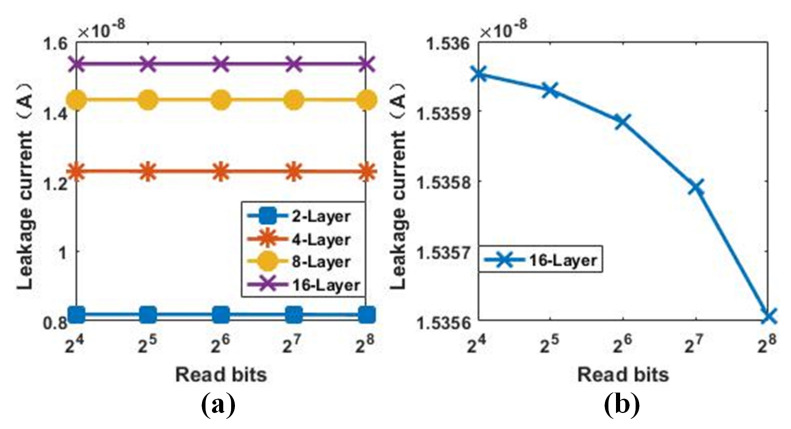
(**a**) The leakage current of multi-bit (from $2^{4}$ to $2^{8}$) parallel read under different layers (from 2 to 16), (**b**) the leakage current under various parallel reading bits when the number of stacked layers is 16 (planar array size is 256×256).

In the read operation, multiple bits can be read in parallel. The relationship between LC and the number of parallel read bits is shown in Figure 10. The number of layers has a much greater impact on the leakage current than the number of bits, as shown in Figure 10a. Figure 10b shows that the leakage current slightly decreases as the number of parallel operation bits increases because, as the selected memory cell increases, the sneak paths in the array decreases. For a 16-layer array, the decrease in the leakage current due to a greater number of selected cells is about 3.5 pA (from $2^{4}$ to $2^{8}$-bits write). However, as the number of parallel operations increases, the operating margin decreases. Therefore, it is essential to trade off the number of bits in the parallel reading. However, Figure 10 suggests that $2^{8}$-bits parallel reading is feasible.

## 4. Conclusions

**Figure 11:**
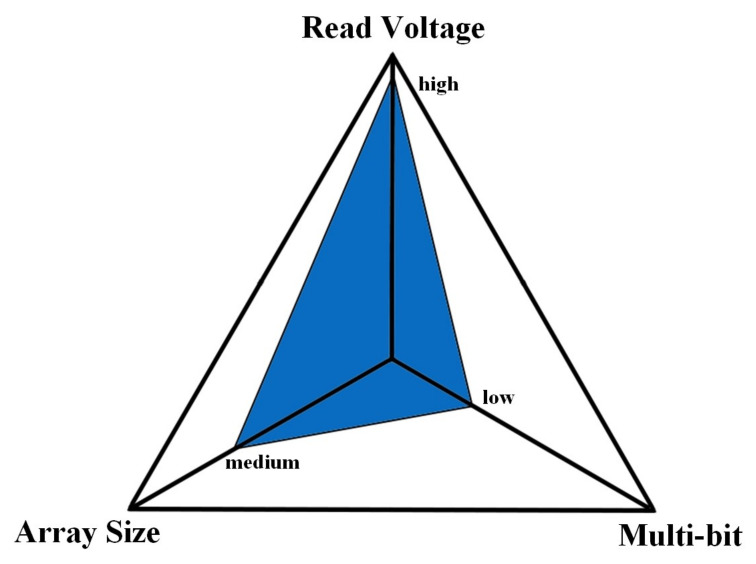
The influence intensity of design parameters on leakage current.

This article analyzes the leakage current of 3-D VRRAM array, which not been fully analyzed in previous research. The influence of the design parameters of the 3-D VRRAM array on the leakage current is summarized in Table 3 and Figure 11. The results show that the growth rate of the leakage current increases as the size of the array increases. Moreover, the operating voltage has a great influence on the leakage current, although a high operation voltage is beneficial to improving the operating margin, the leakage current increases as well, which leads to a decrease in the reliability of the array. Therefore, while ensuring the operation margin, the operation voltage should be reduced as much as possible. Multi-bit operation is an attractive way to decrease the generation of leakage current. It can be seen from the Figure 11 that the read voltage has the greatest influence on the leakage current, and the multi-bit operation has the least influence on the leakage current. Therefore, in the design of the array, it is necessary to minimize the working voltage and to increase the number of parallel operation bits without affecting the function, so that the array can achieve higher performance and lower energy consumption. This paper provides a guideline for the design of a 3-D RRAM array.

## Figures and Tables

**Table 1:** Read and write voltage scheme.

Parameter	Sel-WL	Unsel-WL	Sel-BL	Unsel-BL
Read	$V_{r}$	0	0	0
Write	$V_{w}$	$V_{w}$/2	0	$V_{w}$/2

**Table 2:** Worst-case cell pattens.

Parameter	WL Half-Selected	BL Half-Selected	Unselected
Read HRS	LRS	LRS	LRS
Read LRS	HRS	HRS	LRS
Write	LRS	LRS	LRS

**Table 3:** Summary of the influence of the design parameters on the leakage current.

Parameter	Array Size ↑	Read Voltage ↓	Multi-Bit ↓
LC	↑	↓	↑
